# A study on the correlation between visual–spatial working memory capacity and decision-making performance in 3V2 offensive tactics of soccer players

**DOI:** 10.3389/fpsyg.2026.1866618

**Published:** 2026-06-26

**Authors:** Yanan Zhao, Peng Shi

**Affiliations:** 1 School of Sports Training, Tianjin University of Sport, Tianjin, China; 2 School of Physical Education, Zhengzhou Normal University, Zhengzhou, China; 3 School of Physical Education, Shandong University of Technology, Zibo, China

**Keywords:** object working memory, offensive tactical decision-making, soccer, spatial working memory, visual–spatial working memory capacity

## Abstract

**Objective:**

To explore the correlation between visual–spatial working memory capacity and its subcomponents (object working memory capacity, spatial working memory capacity) and 3V2 offensive tactical decision-making performance in soccer players.

**Methods:**

Sixty male soccer players with national second-class or higher athletic level were selected as participants. The change detection paradigm was used to measure the response time and accuracy of visual–spatial working memory capacity, object working memory capacity, and spatial working memory capacity. A sport specific situational test was applied to evaluate the response time and rationality of 3 V2 offensive tactical decision-making. Participants were divided into high-capacity and low-capacity groups by K-means clustering, and statistical analyses were performed using Mann–Whitney *U* test and partial Spearman correlation analysis.

**Results:**

The high visual–spatial working memory capacity group showed significantly shorter response time (*Z* = −3.944, *p* < 0.001) and significantly lower decision rationality (*Z* = −2.135, *p* = 0.033) in 3 V2 offensive tactical decision-making than the low-capacity group. Similar intergroup differences were observed in object and spatial working memory capacity. After controlling for confounding variables, the response times of visual–spatial, object and spatial working memory capacities were significantly positively correlated with the response time of 3 V2 offensive tactical decision-making (*p* < 0.01) and decision rationality (*p* < 0.05). However, no significant correlations were found between the accuracy of the three working memory types and decision-making performance (*p*>0.05).

**Conclusion:**

The response time of visual–spatial working memory and its subcomponents is a key indicator for predicting 3 V2 offensive tactical decision making performance in soccer players. This study verifies the speed-accuracy trade off effect in sports cognition.

## 1 Introduction

Soccer is an open competitive sport that integrates physical fitness, technical skills, tactical awareness and cognitive ability (Forsman et al., 2016), with the game situation changing rapidly and the pace of local offensive and defensive transitions accelerating constantly. The 3 V2 offensive tactic, as the most common local numerical superiority tactic in matches, is an important means for teams to break down a compact defense and create scoring opportunities (Shi et al., 2023). The effective execution of the 3 V2 offensive tactic not only requires players to possess proficient technical and tactical abilities such as ball control, passing and shooting, but also relies on their ability to quickly extract, process and store visual–spatial information including on-field spatial positions, teammates’ runs and defenders’ positioning within a limited time, and make sound tactical decisions (Clemente et al., 2025; Shi et al., 2023).

With the in-depth application of sport cognitive psychology in the sports field, researchers (Lopes, 2024; Scharfen and Memmert, 2019; Vidigal et al., 2022) have gradually realized that cognitive and psychological ability is an important factor affecting the performance of athletes’ technical and tactical levels. Visual–spatial working memory, as the core cognitive system for athletes to process visual–spatial information and make tactical decisions (Furley and Wood, 2016), plays a crucial role in soccer players during competitions. As an important subsystem of working memory, visual–spatial working memory is mainly responsible for the temporary storage and processing of visual–spatial information (McAfoose and Baune, 2009). Visual–spatial working memory consists of two functional subcomponents: object working memory and spatial working memory (Oliveri et al., 2001). Among them, object working memory refers to the temporary storage and processing of object information, such as the storage and processing of shape and pattern information, which is mainly realized through the “what” occipital-temporal pathway (Li and Chi, 2019). Spatial working memory refers to the temporary storage and processing of spatial information, such as the storage and processing of spatial relations including position and direction, which is mainly realized through the “where” occipital-parietal pathway (Li and Chi, 2019). The capacity of visual–spatial working memory reflects athletes’ ability to temporarily store and process visual–spatial information (Sato et al., 2022), and its level directly influences the efficiency of information processing and the quality of decision-making in competition scenarios (Vaughan and Laborde, 2021).

Furley and Memmert (2012) found that working memory capacity, as controlled attention ability, plays a vital role in athletes’ tactical decision-making process, with athletes with high working memory capacity being better able to concentrate their attention and process complex competition information. In fact, existing studies have confirmed a clear association between visual–spatial working memory capacity and athletes’ decision-making (Li and Chi, 2019; Ye, 2012). Ye (2012) found that the level of visual–spatial working memory capacity had a significant impact on both the accuracy and reaction time of badminton players’ decision-making. Li and Chi (2019) explored the effect of visual–spatial working memory capacity on basketball players’ defensive anticipation tasks and found that participants with a small visual–spatial working memory capacity exhibited faster anticipation reaction speeds. Further research revealed that participants with a small object working memory capacity had faster anticipation reaction speeds, while those with a large spatial working memory capacity achieved higher anticipation accuracy. Cheng and Wang (2026) found that spatial working memory capacity was correlated with the visual search characteristics of tennis players’ offensive tactical decision-making, and the high-capacity group showed lower flexibility in tactical decision-making. Knöllner et al. (2022) identified a potential link between working memory and athletes’ selective attention. Mahfoudh and Zoudji (2022) found that visual–spatial ability is a core cognitive foundation for soccer players’ tactical memory, exerting a direct positive effect on the efficiency of visual processing and memory performance in tactical scenarios, and that athletes with high visual–spatial ability are more likely to benefit from tactical scenarios with low visual realism. In addition, Radhouaned and Akil Abedlkadird (2022) found that methods for improving the visual–spatial ability of soccer strikers can effectively enhance their visual–spatial perception ability. Zhang (2019) found that specialized working memory training can significantly improve the decision-making ability and change detection ability of adolescent soccer players aged 9 to 12.

However, current research on the relationship between visual–spatial working memory capacity and soccer players’ tactical decision-making performance is relatively scarce, especially the specific cognitive research targeting typical local tactics such as 3 V2 offensive play. Moreover, the association between visual–spatial working memory capacity and its subcomponents (object working memory capacity and spatial working memory capacity) and the decision-making performance in 3 V2 offensive tactics has not been clearly defined. Based on this, this study aims to explore the correlation between visual–spatial working memory capacity and soccer players’ decision-making performance in 3 V2 offensive tactics and reveal the inherent relationship between them. It is of great practical significance for enriching the cognitive training theory of soccer and improving the execution effect of the team’s local offensive tactics.

## 2 Methods

### 2.1 Participants

The sample size was calculated *a priori* using G*Power 3.1.9.7 software. Based on previous evidence indicating that skilled players possess significantly higher visual spatial working memory capacity (Valls-Serrano et al., 2022; Xiao and Li, 2026), and combined with effect sizes from similar studies investigating cognitive differences between athletes of varying expertise levels (*ES d* = 0.88; Cheng and Wang, 2026), a test for independent samples was applied with the following parameters: *α* = 0.05, power (1-*β*) = 0.80, and *ES d* = 0.88. The calculation revealed that 34 participants were required (17 skilled players and 17 less-skilled players). To further ensure statistical stability, a supplementary sample size estimation was conducted based on typical effect sizes reported in studies of working memory capacity and soccer decision-making (*ES d* = 1.44; Shi et al., 2022). This calculation indicated that 18 participants would be sufficient. Considering the more conservative estimation based on expertise-related cognitive differences, the final sample size was determined to be 34 participants to ensure adequate statistical power.

The study recruited participants using a convenience sampling method. A total of 60 male players at or above the national second-class level were recruited as participants in this study. In addition, we further verified the rationality of the sample size based on a medium effect size (*r* = 0.4), with *α* = 0.05 and power (1-*β*) = 0.80. The calculation showed that 60 participants were required, which is consistent with the sample size we recruited. All participants were college student players from a university in Liaoning and Shandong Province, aged 18 to 23 years. They possessed solid soccer technical and tactical foundations as well as rich competition experience, with 6 to 10 years of soccer training experience. All of them took part in more than 3 soccer training sessions per week, each lasting for over 30 min, and had all participated in provincial-level soccer competitions. In addition, all participants had normal visual acuity or corrected visual acuity, no visual impairments such as color blindness or color weakness, and no neurological diseases, being able to cooperate in completing all experimental tests. This study was conducted in strict accordance with the Declaration of Helsinki and approved by the Scientific Research Ethics Committee of Shandong University of Technology. All participants signed a written informed consent form prior to the study.

### 2.2 Study design

This study adopted a cross-sectional design to explore the correlation between soccer players’ visual–spatial working memory capacity and their decision-making performance in 3 V2 offensive tactical scenarios. In terms of experimental design, the change detection paradigm (Bassano et al., 2023; Rosen et al., 2014) was used to assess the players’ visual–spatial working memory capacity, spatial working memory capacity and object working memory capacity; a sport-specific situational test was employed to measure their decision-making performance in 3 V2 offensive tactics, with response time and accuracy as the key indicators. In terms of statistical analysis, referring to previous research paradigms (Shi et al., 2022), the K-means clustering algorithm was used to categorize participants into high and low working memory groups based on their visual–spatial working memory capacity, object working memory capacity and spatial working memory capacity respectively, and the 3 V2 offensive tactical decision-making performance was compared between the two groups. On this basis, the study further probed into the correlations of visual–spatial working memory capacity, spatial working memory capacity and object working memory capacity with soccer players’ decision-making performance in 3 V2 offensive tactics.

### 2.3 Variables and measures

The tests of this study were conducted in the Psychology Laboratory of the School of Physical Education, Shandong University of Technology or Liaoning Normal University, which were divided into two parts: the visual–spatial working memory capacity test and the 3 V2 offensive tactical decision-making performance test. The former consisted of three subtests, namely the visual–spatial working memory capacity subtest, the object working memory capacity subtest and the spatial working memory capacity subtest. All tests were carried out in a quiet environment with appropriate lighting. Prior to the tests, the participants were informed of the research objectives, procedures and requirements in detail, and were given practice sessions for the pilot test to ensure that they were familiar with the test operations.

#### 2.3.1 Visual–spatial working memory capacity test

The commonly adopted research paradigm for visual–spatial working memory is the change detection paradigm (Bassano et al., 2023). In this paradigm, participants first encode visual objects or scene stimuli, followed by a blank masking interval, and then judge whether changes exist in the subsequently presented test stimulus arrays. Visual–spatial working memory capacity is measured by gradually increasing the number of stimulus arrays (Rouder et al., 2011). Researchers can flexibly set experimental stimuli, including static arrays, gradual-change videos, event-triggered image sequences and real-life scenarios (Bassano et al., 2023; Rensink et al., 1997; Simons et al., 2000). Based on the core logic of this paradigm, Chinese scholars Li and Chi (2019) developed a change detection measurement tool suitable for exploring athletes’ visual–spatial working memory capacity in the Chinese cultural context, which has been widely recognized and applied in relevant empirical studies (Zhang, 2021). This study mainly recorded the response time and accuracy in the task, and comprehensively determined the participants’ visual–spatial working memory capacity based on these two indicators. The specific measurement procedures are detailed as follows:

##### 2.3.1.1 Visual–spatial working memory capacity subtest

Before the test, the experimenter instructed participants to adjust their sitting posture and seat height to a comfortable position according to the screen, and explained the task instructions. After the test commenced, instructions appeared on the screen prompting participants to press the Spacebar to start the test once they were ready. In addition, instructions were presented before the start of each block, and participants decided for themselves whether to proceed with the test. After participants pressed the Spacebar, a fixation cross “+” appeared at the center of the screen for 500 ms, followed by a 300 ms blank screen. Then, 2 to 6 irregular figures were presented for 300 ms. Participants were required to memorize the shape and position of these irregular figures. Subsequently, another 1,000-ms blank screen was displayed, during which participants were instructed to fixate on the central “+.” After the blank screen, a recognition display was presented (with the same number of figures as the memory display), and participants judged whether the position or shape of any figure had changed. If a change occurred, participants pressed the F key; if no change occurred, they pressed the J key. The test consisted of a practice section and a formal test section. The practice section included 20 trials, with 10 trials for each of the congruent and incongruent conditions. Each condition contained 5 capacity levels, with 2 trials per level. Participants could repeat the practice as needed and proceed to the formal test once they were familiar with the rules. The formal test contained 200 trials, with a capacity range of 2 to 6 items, divided into 5 blocks starting from 2. Each capacity level included 40 trials, with 50% change trials and 50% no-change trials. Participants were allowed a 60-s break between blocks, and the inter-trial interval was 1,000 ms. In addition, test–retest reliability of the measurement was examined in this study. Thirty participants were randomly selected for retesting 2 weeks after the initial assessment. Pearson correlation analysis revealed a high correlation coefficient between the two sets of measurements (*r* = 0.813, *p* < 0.01), indicating that the test had good reliability.

##### 2.3.1.2 Object working memory capacity subtest

Prior to the test, the experimenter instructed participants to adjust their sitting posture and seat height to a comfortable position relative to the screen and explained the task instructions. Once the test began, instructions appeared on the screen prompting participants to press the Spacebar to start the task when ready. In addition, instructions were presented before the start of each block, and participants decided for themselves whether to proceed with the test. After participants pressed the Spacebar, a fixation cross “+” appeared at the center of the screen for 500 ms, followed by a 300-ms blank screen. Then, 2 to 6 irregular figures were presented for 300 ms, and participants were required to memorize the shape of these figures. Subsequently, another 1,000-ms blank screen was displayed, during which participants were instructed to fixate on the central “+.” Following the blank screen, a recognition display was presented (with the same number of figures as the memory display), and participants judged whether the shape of any figure had changed. If a change occurred, participants pressed the F key; if no change occurred, they pressed the J key. The test consisted of a practice phase and a formal test phase. The practice phase included 20 trials, with 10 trials for each of the congruent and incongruent conditions. Each condition comprised 5 capacity levels, with 2 trials per level. Participants could repeat the practice as needed and proceed to the formal test once they were familiar with the rules. The formal test contained 200 trials, with a memory capacity range of 2 to 6 items, divided into 5 blocks starting from 2 items. Each capacity level included 40 trials, with 50% change trials and 50% no-change trials. Participants were allowed a 60-s break between blocks, and the inter-trial interval was 1,000 ms. In addition, the test–retest reliability of the measurement was examined in this study. Thirty participants were randomly selected to be retested 2 weeks after the initial assessment. Pearson correlation analysis revealed a high correlation coefficient between the two measurements (*r* = 0.769, *p* < 0.01), indicating that the test had good reliability.

##### 2.3.1.3 Spatial working memory capacity subtest

Before the test, the experimenter instructed participants to adjust their sitting posture and seat height to a comfortable position relative to the screen and explained the task instructions. Once the test commenced, instructions appeared on the screen prompting participants to press the Spacebar to start the test when ready. In addition, instructions were presented before the start of each block, and participants decided for themselves whether to proceed with the test. After participants pressed the Spacebar, a fixation cross “+” appeared at the center of the screen for 500 ms, followed by a 300-ms blank screen. Then, 2 to 6 irregular figures were presented for 300 ms, and participants were required to memorize the position of these figures. Subsequently, another 1,000-ms blank screen was displayed, during which participants were instructed to fixate on the central “+.” Following the blank screen, a recognition display was presented (with the same number of figures as the memory display), and participants judged whether the position of any figure had changed. If a change occurred, participants pressed the F key; if no change occurred, they pressed the J key. The test consisted of a practice phase and a formal test phase. The practice phase included 20 trials, with 10 trials for each of the congruent and incongruent conditions. Each condition comprised 5 capacity levels, with 2 trials per level. Participants could repeat the practice as needed and proceed to the formal test once they were familiar with the rules. The formal test contained 200 trials, with a memory capacity range of 2 to 6 items, divided into 5 blocks starting from 2 items. Each capacity level included 40 trials, with 50% change trials and 50% no-change trials. Participants were allowed a 60-s break between blocks, and the inter-trial interval was 1,000 ms. In addition, the test–retest reliability of the measurement was examined in this study. Thirty participants were randomly selected to be retested 2 weeks after the initial assessment. Pearson correlation analysis revealed a high correlation coefficient between the two sets of measurements (*r* = 0.834, *p* < 0.01), indicating that the test had good reliability.

#### 2.3.2 3 V2 offensive tactical decision-making performance test

The stimulus materials adopted in this study are all first-person perspective videos of 3 V2 offensive tactical decision-making in real match scenarios restored through live-action shooting. All videos have undergone standardized screening, verification and difficulty control. The original video materials selected in this study are official match recordings of top teams from Europe’s top five leagues during the 2024–2025 seasons, from which authentic and typical 3 V2 local offensive scenarios are extracted, including 6 clips each for left-flank, central and right-flank attacks. In addition, 6 clips of stimulus materials for the preliminary test are prepared, with 2 clips, respectively, for left-flank, central and right-flank attacks. Soccer players with national second class or higher sports athlete grades restored and filmed the selected original videos from the first-person perspective (ball carrier’s perspective) on a standard 11-a-side soccer field. A Canon 6D camera equipped with a 50 mm prime lens was used for shooting, with parameter settings as follows: ISO 100, aperture f/5.6, video resolution of 1920 × 1,080 and frame rate of 50 fps. The shooting height was aligned with the players’ line of sight to ensure the visual perspective was consistent with the actual viewing experience of ball carriers in real matches. A total of 18 videos were finally confirmed for the formal test, including 6 clips each for left-flank, central and right flank attacks. There were 6 practice videos for the pre-test, with 2 clips for each offensive route, aiming to help participants get familiar with the task procedure and response modes. All videos were uniformly edited using Corel Video Studio. The editing rule was that each video starts the moment offensive players initiate off-ball runs or ball-carrying advances, and ends when all players finish their movements. Each video was controlled at approximately 3 s in length to ensure clear scenarios, distinct decision-making points and consistent time pressure.

In terms of scenario construction, all videos present a standard 3 V2 offensive defensive formation. The three offensive players consist of one ball carrier (the shooter) and two off-ball supporting teammates, while the two defensive players are responsible for frontal blocking and lateral auxiliary defense, respectively. All players maintained fixed starting positions. In left-flank scenarios, the ball carrier stood on the left sideline; in central scenarios, the ball carrier positioned himself at the edge of the penalty area in the central zone; in right-flank scenarios, the ball carrier was located on the right sideline. The positions and running directions of off-ball teammates and defenders remained typical and consistent across all scenarios, ensuring stable scene structure and definite tactical implications. To guarantee the validity and reliability of the stimulus materials, three soccer-specific coaches and two sports psychology experts were invited to evaluate the video scenarios in terms of tactical typicality, situational clarity and decision-making difficulty. Final stimulus materials were confirmed after further editing and revision. All videos were ensured to have moderate difficulty, typical scenarios and definite decision options without ambiguity or missing information.

Prior to the formal test, the experimenter projected the videos onto a large screen via a projector, adjusted participants’ sitting posture and viewing distance for clear and comfortable vision, and explained the task procedure, decision-making rules and response methods. Participants first completed six pre-test video trials and proceeded to the formal test after getting familiar with the task. Videos were presented in random order during the test. Participants were required to imagine themselves as on-field ball-carrying attackers and make immediate decisions right after each video ended based on teammates’ movements, defenders’ positions and available space. There were four decision options: passing to nearby teammates, passing to distant teammates, dribbling breakthrough and shooting. Participants reported their decisions verbally, while response time and decision selections were recorded synchronously by the computer. Decision-making response time refers to the duration from the start of video task playback to the moment participants press the key to pause.

Five experts consisting of coaches of the Chinese University Soccer League and national first-class athletes independently evaluated the rationality of participants’ decisions with a five-point scale: 5 points for decisions fully complying with 3 V2 offensive principles, fully leveraging numerical and spatial advantages with extremely low ball-losing risks and high possibility of creating valid shooting or scoring chances; 4 points for decisions in line with relevant offensive principles that make proper use of offensive space with low ball-losing risks to ensure steady ball control and continuous attacks; 3 points for basically reasonable decisions that partially utilize offensive space with moderate ball-losing risks and can sustain attacks with limited threatening effects; 2 points for irrational decisions failing to take full advantage of offensive space with high ball-losing risks and greatly impeding offensive advancement; 1 point for seriously wrong decisions violating 3 V2 offensive principles that waste offensive opportunities and easily result in immediate loss of ball possession. The average value of expert scores was taken as the final rationality score, and intraclass correlation coefficient (ICC) was adopted to test the consistency of expert evaluations, with results showing ICC>0.85 and *p* < 0.01, which demonstrated excellent reliability and credible scoring outcomes.

### 2.4 Statistical analysis

This study used SPSS 21.0 for data processing and statistical analysis. Descriptive statistics were presented as mean (*M*) ± standard deviation (*SD*), as well as frequency and percentage. Firstly, the K-means clustering algorithm was used for participant grouping. Response time and accuracy of visual–spatial working memory capacity, object working memory capacity, and spatial working memory capacity were separately employed as clustering variables, and the number of clusters was set to 2 to divide participants into high-capacity and low-capacity groups for each type of working memory capacity (visual–spatial, object, and spatial). In addition, 10 repeated clustering tests were conducted, and the classification results remained unchanged, demonstrating the stability of the clustering solution. Secondly, the one-sample Kolmogorov–Smirnov test combined with Q-Q plots and P–P plots was used for normality testing. The results showed that the data did not conform to strict normal distribution (see the Results section for detailed outcomes). Therefore, the Mann–Whitney *U* test was adopted to compare the differences in response time and rationality of 3 V2 offensive tactical decision-making between the high-capacity group and low-capacity group. Mean ranks, *Z* statistics and *p* values of Mann–Whitney *U* test were reported in the results. In addition, the effect size *r* for inter-group differences was calculated via the formula *r*= $\frac{|Z|}{\sqrt{N}}$ , where *N* stands for the total sample size. The interpretation criteria of effect size were as follows: *r* < 0.1 represented a trivial effect, 0.1 ≤ *r* < 0.3 a small effect, 0.3 ≤ *r* < 0.5 a moderate effect, and *r* ≥ 0.5 a large effect (Fiel Peres, 2026). In addition, the chi-square test for contingency table was used to compare the composition of athletic grades between groups. Finally, after controlling for confounding variables including age, years of soccer training, athletic level, weekly training frequency, and duration per training session, partial Spearman correlation analysis was performed to examine the correlations between the three types of working memory capacity and 3 V2 offensive tactical decision-making performance. The significance level was set at *p* < 0.05 (significant difference) and the highly significant level at *p* < 0.01 (highly significant difference).

## 3 Results

### 3.1 Basic information of participants

The basic information of the participants is presented in Table 1. The participants had a mean age of 20.35 years (*SD* = 1.70). The mean years of soccer training was 7.98 years (*SD* = 1.42). The average number of training sessions per week was 4.33 (*SD* = 1.13), with a mean duration per training session of 43.83 min (*SD* = 10.91). In terms of athletic level, 24 participants (40.00%) were national second-class athletes, and 36 participants (60.00%) were national first-class athletes.

**Table 1:** Basic information of participants.

Variables	*M*	*SD*	Variables	Freq	%
Age (years)	20.35	1.70	Athletic level		
Years of soccer training (years)	7.98	1.42	National second-class athlete	24	40.00
Number of training sessions per week (sessions)	4.33	1.13	National first-class athlete	36	60.00
Duration per training session (minutes)	43.83	10.91			

K-means clustering divided the 60 players into two groups: a high-capacity group and a low-capacity group. The cluster sizes of the two groups across different indicators are detailed in Table 2 and Table 3. Normality tests were conducted on the data of visual–spatial, object and spatial working memory capacity. All *p* values were less than 0.01, indicating non-normal distribution. The Mann–Whitney *U* test was adopted for inter-group comparison. No significant intergroup differences in age, gender, years of soccer training, weekly training frequency and single-session training duration were observed between the high and low visual–spatial working memory capacity groups (*p* > 0.05). For the object and spatial working memory subgroups, age, gender, years of soccer training and weekly training frequency were comparable across high and low-capacity groups (*p* > 0.05), whereas participants in the high capacity groups spent significantly longer on each training session (*Z* = -0.351, *p* < 0.001).

**Table 2:** Results of intergroup comparison between the high and low visual–spatial working memory capacity groups.

Variables	High-capacity group		Low-capacity group		*Z*	*P*
	*M* ± *SD*	Mean rank	*M* ± *SD*	Mean rank		
Age (years)	20.06 ± 1.47	27.99	20.76 ± 1.92	34.02	−1.345	0.179
Years of soccer training (years)	8.09 ± 1.44	31.77	7.84 ± 1.41	28.72	−0.682	0.495
Number of training sessions per week (sessions)	4.34 ± 1.03	30.81	4.32 ± 1.28	30.06	−0.171	0.864
Duration per training session (minutes)	44.57 ± 10.67	31.77	42.80 ± 11.37	28.72	−0.692	0.489

Athletic level	Freq.	*%*	Freq.	*%*	*χ^2^*	*p*
National second-class athlete	21	60.0%	10	40.0%	2.336	0.126
National first-class athlete	14	40.0%	15	60.0%		

**Table 3:** Results of intergroup comparison between the high and low object or spatial working memory capacity groups.

Variables	High-capacity group		Low-capacity group		*Z*	*p*
	M ± SD	Mean rank	M ± SD	Mean rank		
Age (years)	20.16 ± 1.54	28.88	20.82 ± 2.01	34.59	−1.162	0.245
Years of soccer training (years)	8.00 ± 1.46	30.70	7.94 ± 1.35	30.00	−0.142	0.887
Number of training sessions per week (sessions)	4.37 ± 1.09	31.15	4.24 ± 1.25	28.85	−0.476	0.634
Duration per training session (minutes)	46.98 ± 11.03	35.33	35.88 ± 5.07	18.29	−0.351	<0.001

Athletic level	Freq.	*%*	Freq.	*%*	*χ^2^*	*p*
National second-class athlete	23	53.5%	13	76.5%	2.681	0.102
National first-class athlete	20	46.5%	4	23.5%		

### 3.2 Grouping of visual–spatial working memory capacity

K-means clustering divided the 60 players into two groups: a high-capacity group and a low-capacity group. The cluster sizes of the two groups across different indicators are detailed in Table 4. Normality tests were conducted on the data of visual–spatial, object and spatial working memory capacity. All *p* values were less than 0.01, indicating non-normal distribution. The Mann–Whitney *U* test was adopted for inter-group comparison. Further inter-group comparison revealed that in visual spatial working memory capacity, the high-capacity group exhibited significantly faster response time than the low-capacity group (*Z* = −6.560, *p* < 0.001) with a medium large size (*r* = 0.847), while no significant difference was found in accuracy (*Z* = −1.920, *p* = 0.055). For object working memory capacity, the high capacity group had markedly shorter response time (*Z* = −5.996, *p* < 0.001) with a small large size (*r* = 0.774), and accuracy showed no statistical difference (*Z* = −0.459, *p* = 0.646). In spatial working memory capacity, the high-capacity group also presented significantly faster response time (*Z* = −5.996, *p* < 0.001) and a small large size (*r* = 0.775), with insignificant accuracy differences (*Z* = −0.328, *p* = 0.743). In conclusion, individuals with high visual–spatial working memory capacity achieve quicker responses, indicating reasonable grouping classification.

**Table 4:** Results of K-means clustering test for visual–spatial working memory capacity, object working memory capacity, and spatial working memory capacity.

Variables	High-capacity group			Low-capacity group			*Z*	*P*	*r*
	*n*	*M* ± *SD*	Mean rank	*n*	M ± SD	Mean rank			
Visual–spatial working memory capacity response time (ms)	35	1310.14 ± 159.37	18.00	25	1556.00 ± 169.40	48.00	−6.560	<0.001	0.847
Visual–spatial working memory capacity accuracy (%)	35	82.99 ± 5.94	26.84	25	86.80 ± 7.76	35.62	−1.920	0.055	0.248
Object working memory capacity response time (ms)	43	1224.42 ± 173.28	22.00	17	1515.35 ± 138.27	52.00	−5.996	<0.001	0.774
Object working memory capacity accuracy (%)	43	85.71 ± 7.47	31.15	17	85.00 ± 5.05	28.85	−0.459	0.646	0.059
Spatial working memory capacity response time (ms)	43	1185.26 ± 182.87	22.00	17	1511.88 ± 140.98	52.00	−5.996	<0.001	0.774
Spatial working memory capacity accuracy (%)	43	86.34 ± 7.04	30.97	17	86.00 ± 5.05	29.32	−0.328	0.743	0.042

### 3.3 Intergroup comparative analysis of 3 V2 offensive tactical decision-making performance among players with different memory capacities

Normality tests were performed on tactical decision response time and rationality data. All *p* values were less than 0.01, suggesting non-normal distribution. The Mann–Whitney *U* test was used for inter-group comparisons. Consistent classification data of object and spatial working memory capacity were yielded in K-means clustering analysis, resulting in generally identical outcomes. As shown in Table 5, the high visual–spatial working memory capacity group had significantly shorter response time in 3 V2 offensive tactical decision-making (*Z* = −3.944, *p* < 0.001) with a small large size (*r* = 0.509), and notably lower decision rationality (*Z* = −2.135, *p* = 0.033, *r* = 0.036). For the high object working memory capacity group, response time was remarkably faster (*Z* = −2.174, *p* = 0.030, *r* = 0.281), while decision rationality was significantly lower (*Z* = −2.279, *p* = 0.023, *r* = 0.294). The high spatial working memory capacity group shared the same statistical results: faster response time (*Z* = −2.174, *p* = 0.030, *r* = 0.281) and reduced decision rationality (*Z* = −2.279, *p* = 0.023, *r* = 0.294). In conclusion, athletes with higher working memory capacity achieve quicker responses yet exhibit lower decision rationality. This finding reflects the typical speed-accuracy trade-off characteristic prevalent in the exercise-cognition field; accordingly, superior overall tactical decision-making performance cannot be simply inferred from higher processing capacity alone.

**Table 5:** Results of intergroup comparative analysis on 3V2 offensive tactical decision-making performance of players with different memory capacities

Variables	Visual-spatial working memory capacity						*Z*	*p*	*r*
	High-capacity group			Low-capacity group					
	*n*	*M*±*SD*	Mean rank	*n*	*M*±*SD*	Mean rank			
Response time (ms)	35	1030.29±118.73	22.99	25	1294.00±304.80	41.02	−3.944	<0.001	0.509
Rationality (score)	35	3.93±0.60	26.44	25	4.26±0.39	36.18	−2.135	0.033	0.276

Variables	Object working memory capacity						*Z*	*p*	*r*
	High-capacity group			Low-capacity group					
	*n*	*M*±*SD*	Mean rank	*n*	*M*±*SD*	Mean rank			
Response time (ms)	43	1091.63±169.31	27.42	17	1262.94±367.56	38.29	−2.174	0.030	0.281
Rationality (score)	43	3.96±0.58	27.28	17	4.34±0.31	38.65	−2.279	0.023	0.294

Variables	Spatial working memory capacity						*Z*	*p*	*r*
	High-capacity group			Low-capacity group					
	*n*	*M*±*SD*	Mean rank	*n*	*M*±*SD*	Mean rank			
Response time (ms)	43	1091.63±169.31	27.42	17	1262.94±367.56	38.29	−2.174	0.030	0.281
Rationality (score)	43	3.96±0.58	27.28	17	4.34±0.31	38.65	−2.279	0.023	0.294

### 3.4 Correlation between working memory capacity and 3 V2 offensive tactical decision-making performance

After controlling for confounding variables including age, athletic level, years of soccer training, weekly training frequency and single-session training duration, the reaction times of visual–spatial, object and spatial working memory capacity were all significantly positively correlated with decision-making response time (*p* < 0.01). Similarly, these three indicators of response time showed significant positive correlations with decision-making rationality (*p* < 0.05). In addition, the accuracy of visual–spatial, object and spatial working memory capacity had no significant correlations with decision-making response time (*p* > 0.05), nor were significant correlations detected between the three types of working memory accuracy and decision-making rationality (*p* > 0.05). Overall, slower processing speed of working memory capacity in soccer players corresponds to poorer performance in decision speed and decision rationality, whereas the accuracy of working memory cannot predict the two indicators of tactical decision-making (Table 6).

**Table 6:** Results of correlation analysis between visual-spatial working memory capacity and 3V2 offensive tactical decision-making performance.

Variables	3V2 offensive tactical decision-making	
	Response time (ms)	Rationality (score)
Visual-spatial working memory capacity response time (ms)	0.628**	0.366*
Visual-spatial working memory capacity accuracy (%)	−0.031	−0.209
Object working memory capacity response time (ms)	0.623**	0.288*
Object working memory capacity accuracy (%)	−0.091	−0.145
Spatial working memory capacity response time (ms)	0.605**	0.285*
Spatial working memory capacity accuracy (%)	−0.049	−0.244

*p<0.05; **p<0.01.

## 4 Discussion

### 4.1 Discussion of main findings

This study adopted the change detection paradigm and sport-specific situational tests to systematically explore the correlations between visual–spatial working memory and its two core subcomponents (object and spatial working memory capacity) and the response time and rationality of soccer players’ decision-making in 3 V2 offensive scenarios. It reveals the association between cognitive resource characteristics and soccer tactical decision-making behaviors, providing empirical evidence for interpreting the underlying cognitive logic of soccer players’ tactical decision-making.

This study indicated that the response times of visual–spatial, object and spatial working memory capacity were significantly positively correlated with both decision making latency and decision rationality in soccer players’ 3v2 offensive tactical tasks. Overall, longer response time of visual–spatial working memory capacity led to prolonged tactical decision-making time yet markedly improved decision rationality, verifying the speed-accuracy trade off effect in sports cognition (Donkin et al., 2014; Spieser et al., 2017). Intergroup comparisons between high and low working memory capacity groups further validated this effect. The high-capacity group exhibited remarkably faster decision response time but significantly lower decision rationality compared with the low capacity group.

The results of the present study are consistent with those reported by Shi et al. (2023) and Glavaš et al. (2023), who indicated that individuals with higher spatial working memory capacity may exhibit relatively lower tactical decision-making performance. In the dynamic and competitive 3 V2 context, visual–spatial information such as field space, teammates’ movement trajectories, and defenders’ positions changes continuously and intricately. Object working memory is responsible for processing concrete visual information including players, the ball and field markers, while spatial working memory deals with spatial relational information such as relative positioning, movement paths and offensive spatial arrangement (Monfort et al., 2019; Oliveri et al., 2001). A longer processing duration of visual–spatial working memory enables soccer athletes to allocate sufficient cognitive resources to comprehensively capture, screen and integrate diverse spatial and object cues on the pitch. This allows for in-depth prediction of defenders’ movement tendencies, evaluation of the advantages and disadvantages of offensive options, and avoidance of judgment biases caused by impulsive decision-making, thereby ensuring the scientificity and rationality of tactical decisions (Furley and Memmert, 2015; Heisler et al., 2023; Shi et al., 2023). In contrast, athletes with faster working memory processing and shorter reaction times can make tactical selections rapidly with better decision agility; however, their information processing tends to be superficial. Such individuals are prone to neglect critical situational details and fail to fully weigh the risks and benefits of alternative offensive strategies, which ultimately reduces decision-making rationality (Cheng and Wang, 2026; Shi et al., 2023). In conclusion, it cannot be one-sidedly assumed that superior visual–spatial working memory capacity comprehensively improves 3 V2 tactical decision-making; the processing speed of players’ working memory produces a trade-off between decision speed and decision quality.

However, this study found no significant correlation between the accuracy of visual–spatial working memory capacity and 3 V2 offensive tactical decision-making performance. The primary reason is that working memory accuracy measured by the change detection paradigm primarily reflects the efficiency of basic cognitive processing under static, standardized laboratory conditions, which is divorced from the specialized soccer environment characterized by high-intensity confrontation, dynamic situational changes, and intense time pressure. Soccer tactical decision making does not merely rely on the accuracy of basic memory recognition; instead, it places greater demands on individuals’ capabilities in resource allocation, real-time information updating, and context-adaptive processing amid complex dynamic information (Glavaš et al., 2023; Romeas et al., 2016). Accurate recognition based on basic working memory cannot be directly translated into superior tactical judgment in high-intensity competitive scenarios (Romeas et al., 2016). This explains why memory accuracy fails to effectively predict sport-specific tactical decision-making outcomes. Meanwhile, it suggests that in research on exercise cognition, the compatibility between general cognitive indicators and sport-specific behaviors should be evaluated dialectically according to the characteristics of individual sports.

### 4.2 The main value of this study

From a theoretical perspective, this study systematically verifies that the response time of visual–spatial working memory and its subcomponents is significantly correlated with the response time and rationality of decision-making in the typical 3 V2 offensive scenarios of soccer, enriching the theoretical system of soccer cognitive psychology. This study verifies the speed-accuracy trade-off effect in the sports domain, explains the inherent relationship between working memory processing duration and decision-making quality, and provides empirical evidence for understanding the cognitive mechanism of tactical decision-making in soccer players. Furthermore, this study demonstrates that working memory accuracy measured in laboratory settings is difficult to predict on-field decision-making performance, emphasizing that general cognitive indicators should be applied dialectically in combination with sport-specific characteristics, thus providing methodological references for future research on sport-specific cognition.

From a practical perspective, this study provides directions for optimizing soccer tactical training: in scenarios requiring quick counterattacks and high-pressure pressing, cognitive training can be used to shorten working memory response time and improve decision-making response time; in positional attack and tight-space breakthrough situations, athletes can be guided to appropriately slow down their processing pace to enhance decision-making rationality. In addition, this study provides a basis for cognitive intervention training, namely that targeted visual–spatial working memory training programs can be developed to improve athletes’ information integration and decision-making ability in small-sided offensive situations such as 3 V2.

### 4.3 Limitations of this study

Although this study confirmed the correlation between visual–spatial working memory capacity and 3 V2 offensive tactical decision-making performance in soccer players, it has certain limitations. Firstly, this study used the change detection paradigm to measure working memory capacity in a static and standardized laboratory environment, which differs greatly from the real scenario of soccer matches characterized by high-intensity confrontation, dynamic changes, and intense time pressure. As a result, laboratory indicators are difficult to effectively predict actual on-field decision-making performance. Secondly, this study is a cross-sectional correlational study, which can only reveal the correlational relationship between variables but cannot verify the causal relationship between visual–spatial working memory capacity and tactical decision-making performance, nor clarify the direction of their effects. Finally, participants were only male football athletes with Grade 2 national certification or above from one university in Shandong Province and one in Liaoning Province. The sample presented high homogeneity in source, age and competitive level. Hence, the research conclusions cannot be generalized to female, adolescent, amateur, professional players or athletes from other regions. In addition, the K-means clustering based on object and spatial working memory data resulted in a marked imbalance in sample size between high and low-capacity groups. Such imbalance stemmed from the natural distribution of the original participant data; nonetheless, the large discrepancy in group sample sizes may partially limit the reliability of between-group comparison outcomes.

Future research can be improved to address the deficiencies of this study. In terms of measurement paradigms, dynamic situational tasks closer to real soccer competition can be adopted to enhance the predictive power of cognitive indicators for on-field decision-making. In terms of research design, longitudinal intervention experiments can be conducted to further verify the causal association between visual–spatial working memory capacity and tactical decision-making performance through targeted working memory training, and clarify the actual effect of cognitive training on improving tactical decision-making ability. In terms of participant recruitment, future studies should expand sampling to include soccer players across diverse ages, competitive levels and regions to improve the generalizability of findings. Meanwhile, pre-experiment sample design can be optimized to avoid severe sample imbalance after clustering. In addition, future studies can integrate multi dimensional cognitive and behavioral variables such as attention control, sports experience, and tactical understanding to more comprehensively reveal the multiple influencing mechanisms of tactical decision-making in soccer players, so as to provide a more systematic and practically valuable theoretical basis for scientific talent selection, cognitive training, and tactical ability improvement of soccer players.

## 5 Conclusion

In this study, the change detection paradigm and sport-specific situational tests were used to investigate the correlation between visual–spatial working memory capacity and its subcomponents (object working memory and spatial working memory) and decision-making performance in the 3 V2 offensive tactics of soccer players. The results revealed that the response times of visual–spatial, object and spatial working memory capacities were significantly positively correlated with both the response time and rationality of 3 V2 offensive tactical decision-making. In addition, the accuracy of the three types of working memory capacity had no significant correlation with decision-making performance. This study verifies an evident speed-rationality trade-off effect: superior working memory capacity cannot optimize decision speed and decision rationality simultaneously and does not equate to better overall tactical decision-making performance. Meanwhile, static cognitive indicators in the laboratory are difficult to effectively predict actual decision-making performance in the dynamic and confrontational context of soccer, highlighting the importance of matching indicators with sport-specific scenarios in sports cognition research.

## Data Availability

The raw data supporting the conclusions of this article will be made available by the authors, without undue reservation.
